# Why Pakistani Women Do Not Use Intrauterine Contraceptive Devices: A Systematic Review of Barriers and Misconceptions

**DOI:** 10.7759/cureus.47378

**Published:** 2023-10-20

**Authors:** Waseem Sajjad, Khadija Ishaq, Sunaina Asghar

**Affiliations:** 1 Department of Community Medicine and Public Health, Mayo Hospital, Lahore, PAK

**Keywords:** ectopic pregnancy, maternal health, community health concerns, contraception, family planning methods, intrauterine contraceptive devices

## Abstract

This review explores barriers limiting the adoption of Intrauterine Contraceptive Devices (IUCDs) in Pakistan, focusing exclusively on local articles. As Pakistan's high population calls for widespread contraception, we aim to pinpoint obstacles hindering IUCD utilization, irrespective of parity.

We conducted a comprehensive search of PubMed, Google Scholar, PakMedinet, and Wiley Online Library for English-language primary studies published between 2000 and 2022, reporting on IUCD utilization in Pakistan.

Our analysis reveals multiple barriers impeding IUCD use in Pakistan. These encompass patriarchal social norms, male dominance, low education, socioeconomic status, and unemployment. Post-insertion health concerns, inadequate counseling, government commitment, and awareness were also identified barriers. Provider confidence, client trust, women's autonomy, social constraints, and limited male partner involvement hindered IUCD adoption. A desire for larger families and male offspring, vague religious beliefs, fear, and misconceptions further restricted usage. Accessibility and high service costs also posed challenges.

This review highlights prevailing impediments to IUCD adoption in Pakistan, encompassing knowledge gaps, motivation deficits, resistance from husbands and in-laws, cultural and religious beliefs, limited access, and communication barriers. To promote IUCDs as a modern contraceptive method, it is essential to raise awareness among both men and women. Active involvement of religious leaders and community stakeholders is crucial in addressing these social factors hindering IUCD utilization.

## Introduction and background

Pakistan, being a developing nation is struggling with a lot of problems. Besides political unrest, economic stress, post-COVID effects, lack of educational facilities, and poor literacy rate, the increasing population is a problem of serious concern. According to the Pakistan Bureau of Statistics (PBS), the latest census (2017) reported the population growth rate of Pakistan to be 2.4% during the intercensal period since 1998 [1]. With this pace, Pakistan is going to be the fifth most populous country in the world by 2050 [1,2]. Despite such huge population growth, the corresponding measures to control it are declining. In terms of Couple Years of Protection (CYP) for the year 2019-20, the Overall Contraceptive Performance is -8.3% based on Family Planning Service Statistics (FPSS) data collected from various departments [3]. Considering method-wise comparison by the Department of Health (DoH), a substantial decrease has been depicted in the use of intrauterine contraceptive devices (IUCDs) i.e. -7.0% for 2019-20 in comparison to 2018-2019 [3].

Rapid population growth can lead to the exacerbation of unemployment and a scarcity of health and education resources [4]. Also, low birth spacing, besides a characteristic of a drastic population rise, poses a greater risk for maternal and infant health issues [5]. Studies link a small interval between pregnancies to an increased probability of stillbirth, premature birth, and low weight at birth along with a severe impact on the mother’s health [6]. Children born after 6 months of previous pregnancy are 60% not liable to survive [7]. To overcome these issues, the use of contraceptives is encouraged, preventing 54 million unintended pregnancies, and 26 million abortions, making a total prevention of 79000 maternal deaths and 1.1 million infant deaths, but still, the contraceptive prevalence rate (CPR) of our country is just 35.4% owing to poor family planning strategies [8]. LARCs (Long Acting Reversible Contraceptives) such as intrauterine contraceptive devices provide harmless and effectual protection against unwanted pregnancies for a long time with the additional benefit of very minor user involvement [9].

Frequently cited (albeit with subtle misdirection) is the fact that intrauterine devices (IUDs) hold the title as the most widely utilized reversible form of contraception globally. No doubt this claim can largely be attributed to countries like China which accounts for 60% of the overall use of IUDs in the world [10]. Although given the efficiency and safety of IUDs, they are the most used LARC all over the world [11]. But the fact of attention is that almost all countries are struggling with the proper implementation of the IUDs in their domain. Financial, religious, lack of awareness, poor counseling, and insufficiently trained workers for the delivery of these services to the public are the major prevailing concerns worldwide [10-14]. In such a worldwide circumstance, Pakistan is no exception, but still, some specific barriers are prevailing regarding the utilization of IUDs as LARC. 

Pakistan, being a highly populated country, is focused on extensive family planning and contraceptive use. However modern and long-acting contraceptives like IUDs are poorly practiced. What are the barriers that impede the widespread use of these modern contraceptive measures, the IUDs? Our primary aim of this study is to discern these impediments and misconceptions that restrict the broader adoption of IUCDs, with a particular emphasis on apprehensions prevailing in Pakistan. It is worth mentioning that a significant portion of these barriers apply to the employment of IUCDs in all women, irrespective of their parity. It is believed that this study makes several novel contributions to the existing literature. Firstly, while prior studies have addressed the barriers to contraceptive use in Pakistan, our research focuses specifically on IUDs, which are an underutilized and highly effective form of contraception. By exclusively examining this method, we were able to provide in-depth insights into the specific barriers and misconceptions surrounding IUD use, thereby addressing a significant knowledge gap in the field. Secondly, this review not only identifies the barriers and misconceptions but also proposes potential strategies to overcome them. By analyzing the common themes and recommendations emerging from the reviewed studies, we have developed a set of evidence-based recommendations that could facilitate the promotion and uptake of IUDs in Pakistan. It’s a strong hope and belief that these practical suggestions will be valuable for policymakers, healthcare providers, and public health professionals working in family medicine and community health.

## Review

Materials and methods

We conducted a comprehensive search of the literature by using PubMed, Medline, Google Scholar, Wiley Online Library, and PakMediNet to find articles written in English between 2000 and 2020 with a special focus on those papers that published Pakistani data. The search was performed using specific MeSH headings: contraception, family planning; intrauterine devices; copper; intrauterine devices, medicated; attitudes; satisfaction; cost-effectiveness; efficacy; and clinician knowledge. We conducted a comprehensive search for primary studies, encompassing various study designs, that examined the obstacles or challenges related to the utilization of IUCDs within the context of Pakistan. To identify additional pertinent studies, we reviewed the reference lists of all the articles. However, due to constraints in resources, we opted not to carry out a search of the grey literature or hand-searching. Additional articles that evaluated the clinical outcomes of intrauterine contraceptives (IUCs) were analyzed, with a specific emphasis on case reports, cohort studies, and clinical trials wherever feasible. Our objective was to investigate the barriers within the healthcare system, healthcare providers, and users that affect the adoption of IUCs, and to examine potential approaches to mitigate these obstacles in Pakistani societal and demographic contexts.

**Figure 1:**
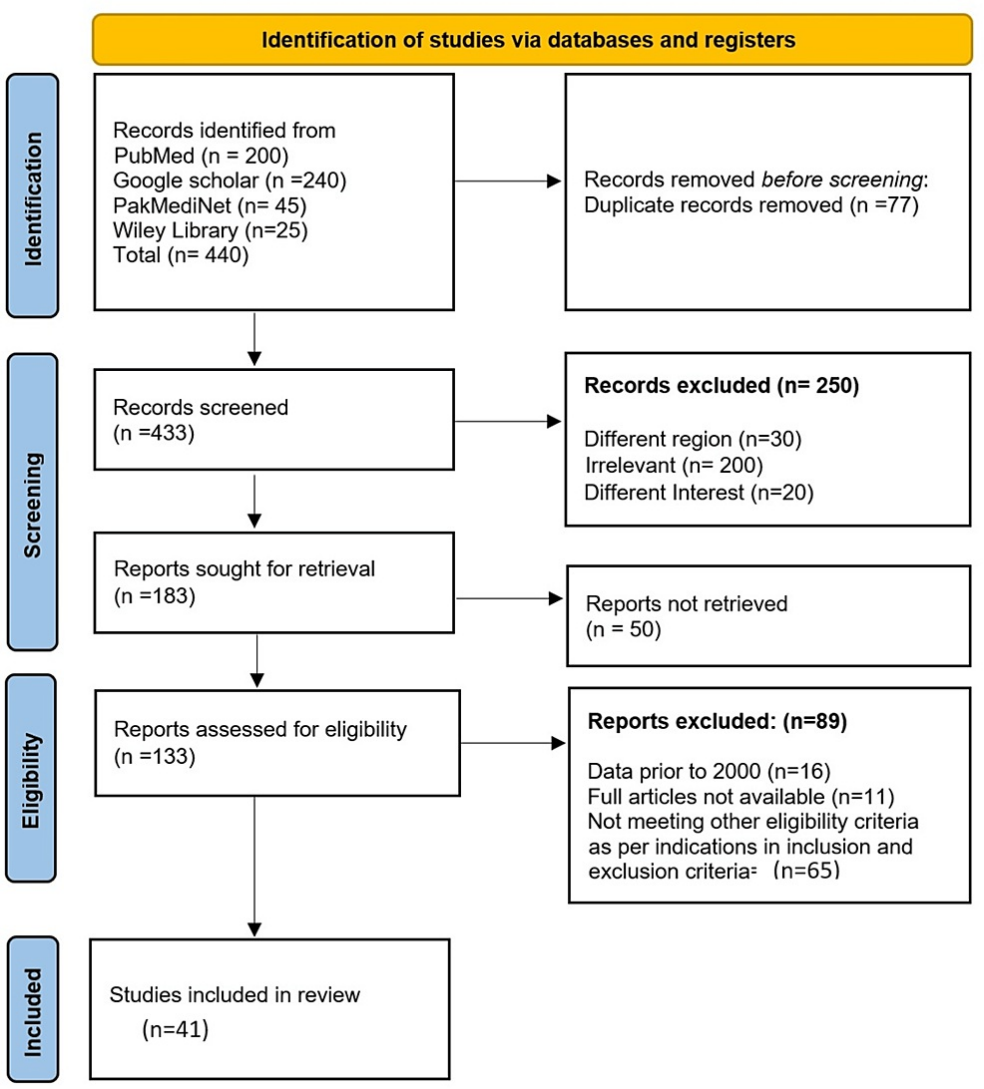
PRISMA flow diagram This flow diagram visualises the process of searching for literature and different stages till the selection of the final ones. PRISMA: Preferred Reporting Items for Systematic Reviews and Meta-Analyses

The risk of personal bias was reduced by independent screening by each author, which was later subjected to group discussion in the presence of a mediator who was not part of the research project, to make it completely unbiased, and each and every aspect of the studies was evaluated before including them in the systematic review. Preferred Reporting Items for Systematic Reviews and Meta-Analyses (PRISMA) guidelines were strictly followed throughout the study and manuscript writing by following a narrative approach (Figure 1).

Inclusion and Exclusion Criteria

Robust selection criteria were introduced to attain the desired objectives and pertinent findings. The inclusion criteria thus finalized were as follows.

Population: Studies involving Pakistani women of reproductive age were included (18-49 years).

Study Design: There were no restrictions on the study design, however, primary research studies (quantitative, qualitative, or mixed-methods studies) were the main center of focus.

Language: Only studies published in the English language were considered.

Setting: Studies conducted in Pakistan or among Pakistani women living in other countries were included.

Intervention: Studies focusing on IUCDs as the main contraceptive method were included.

Outcomes: Studies reporting barriers, myths, misconceptions, or factors influencing the use of IUDs among Pakistani women were included.

Timeframe: All the studies included were those published between 2000 and 2022. Only those full-text articles were included which were accessible for free for the readers.

Along with the inclusion criteria, exclusion criteria were also made clear to make the screening process easy and hassle-free. The criteria were as follows.

Population: Studies involving women from countries other than Pakistan were excluded.

Study Design: Editorials, commentaries, conference abstracts, and letters were excluded.

Language: Studies published in languages other than English were excluded.

Setting: Studies conducted exclusively among specific subpopulations (e.g., women with specific medical, surgical, or environmental conditions) were excluded unless they provided relevant insights into the broader Pakistani population.

Intervention: Studies that do not focus primarily on IUDs or include other contraceptive methods without specific emphasis on IUDs were excluded.

Outcomes: Studies that did not report barriers, misconceptions, or factors influencing the use of IUDs among Pakistani women were excluded.

Duplicate Studies: In the case of multiple publications based on the same study, only the most comprehensive and recent publications were included. Grey literature and hand-searching were omitted due to financial constraints and access. All the paid literature was omitted because of financial constraints.

For sentence structure and language assistance, online Grammarly (Grammarly, Inc., San Francisco, USA), and QuillBot (QuillBot, Chicago, USA) were used. Zotero Reference Manager (Corporation for Digital Scholarship, Vienna, USA) was used for citation and reference writing and literature library management during the course of the study. 

Results

The results extracted from this study are as follows. They are summarized and their integration and observed overlapping nature are illustrated in the table at the end of this section.

Patriarchal Social Behavior and Male Dominance

In Pakistan, the use of IUDs is influenced by the decision of the male partner. Many women were found to be willing to use Postpartum Intrauterine Devices (PPIUDs), but after discussing with their male spouse only half of the willing ones remained constant with their decision [14,15]. Moreover, a study showed that of all the women who discontinued IUD use, more than two-thirds did because of the husband or in-laws' influence [16,17]. It’s not merely a matter of IUDs, the same is true for simple family planning methods of any kind to somewhat varying extents [18].

Low Education, Socioeconomic Status, and Unemployment (in Women)

Studies reveal that the use of PPIUDs is more accepted among women of higher socioeconomic and employed status [18]. Unfortunately, there is a poor trend in women's employment and education in Pakistan and a study reveals a higher IUD utilization rate among educated women as compared to uneducated [6,19]. Adding to this a vast population belongs to low economic status. According to the Asian Development Bank, 21.9% population is below the national poverty line [20]. According to the World Bank, women constitute only 23.3% of the total workforce [21]. Moreover, the financial barriers also add to this i.e. a research finding indicates that patients who self-financed their contraceptive devices had a higher likelihood of discontinuing usage within six months, in contrast to those who received vouchers for intrauterine devices (IUDs) [9].

COVID-19, a Negative Effect On IUCDs

Amid the pandemic, the usage of IUCDs experienced a decrease, while the popularity of condoms remained steady. Nonetheless, client satisfaction with the provision of services through both channels remained satisfactory [22]. Moreover, COVID-19 has affected overall contraception worldwide and Pakistan is no exception [23].

Post-Insertion Health Concerns

Though the IUDs are one of the safest long-term contraceptive measures and the complications are very rare, it is still necessary to make the client aware of the possible complications. To avoid or tackle it in time, the client must be educated about self-examination of the threads [24]. Cases with perforations and pelvic abscesses are, though, rare but reported in Pakistan, with the main issue of the client's lack of knowledge of self-examination [25,26]. Some also complained about pain, excessive bleeding, even related to obesity and hypertension, and infecundity [16,19]. Nevertheless, the overall infection rate due to IUDs is not exceeding 1.3% [27]. A cohort study revealed that about half of the women who discontinued IUDs had health concerns like excessive or irregular bleeding and pain [16].

Poor Counseling Regarding IUDs

There is poor counseling in hospitals and Primary Health care Centers (PHCs] for the promotion of the use of IUDs. Studies show that among counseled women (post-birth or miscarriage or abortion) only 2.2% accepted the use of IUCDs [27]. Lack of or inadequate counseling has developed myths and fears about IUDs’ insertion especially immediate PPIUDs’ insertion [17,28,29]. The insufficient information provided regarding contraceptive methods including IUCDs has led to the inability to attain the desired results [30]. Additionally, women who possess limited knowledge about family planning are more prone to discontinuing contraception use as a result of experiencing adverse effects or encountering failure with their chosen contraceptive method [31].

Poor Government Dedication and Lack of Awareness Regarding IUDs

Even though IUDs have been accessible in Pakistan for several decades, recent research conducted on married women in Punjab indicates that merely about one-third of them were aware of IUDs [32,33]. An experimental study showed that out of 7314 women who were counseled during the study, only 8.12% had ever heard about the IUDs and none of them ever adopted IUDs [34]. An essential aspect contributing to the lack of effective promotion of intrauterine devices (IUDs) in Pakistan is the inadequate commitment of the government to providing comprehensive education, logistics, and national-level support for IUD-related initiatives. This deficiency in government efforts hinders the successful promotion and uptake of IUDs as a contraceptive option at the national level [10]. This is constant with the fact that family planning intervention in rural areas in comparison to urban is less which is evident by increased total fertility rate (TFR) and unfulfilled needs in the countryside [35]. In numerous regions of Pakistan, the prevalent practice of early marriages often results in a dearth of basic education among women. Consequently, these women are inadequately informed about contraceptive methods, which in turn contributes to the occurrence of unintended pregnancies due to the absence of effective family planning measures [36].

Lack of Provider’s Confidence and Client Trust

There are various plausible reasons for the relatively low rates of utilization of the PPIUD such as diminished confidence among healthcare providers, hesitancy or mistrust among clients towards the method, as well as systemic obstacles related to the management of contraceptive supply chains [37]. Moreover, studies have found that physicians favor techniques that require less time and work as compared to IUDs [14]. Research indicates a concerning need for a substantial number of qualified and skilled female healthcare providers at local health units to handle LARCs like IUDs instead of relying solely on periodic camps organized by public or private organizations. This highlights the crucial importance of having trained healthcare providers readily available at the local level to ensure the safe and effective provision of LARC methods [38].

Poor Women's Autonomy and Social Restrictions

The intricate interplay between society and culture exerts a profound influence on contraceptive utilization among populations. Within the confines of the home, women face the constraint of purdah, a cultural practice that restricts their mobility and hinders their ability to access vital information on contemporary contraceptive methods available at healthcare centers [39-41]. Moreover, in joint families, it has been observed that women's decision is subjected to the family decision, especially the mother-in-law who owns the authority for the recommendation of the family planning method, and the use of IUDs is not an exception [42].

Poor Involvement of the Male Partner in FPPs (Family Planning Programs)

Family planning in Pakistan primarily focuses on women due to cultural and societal perceptions of gender roles, limited access to education and economic opportunities for women, and a healthcare system that primarily caters to women's reproductive health needs. This attitude has created a lack of interest and non-involvement of men in family planning programs including IUD utilization leading to poor knowledge and health concerns of their female partner that is freezing IUD utilization [43]. Such programs with uneven focus have failed to attain the targeted goals [44]. An interesting fact has been unveiled by a study, which indicated that men prefer a bigger family linking it with prosperity and strength while on the contrary women urge to have smaller families with better standards of life [45]. This highlights the inevitable involvement of male partners in FPPs.

Desires for Larger Family Size and a Preference for Male Offspring

A recent study assessed the impact of the number of children on the utilization and discontinuation rates of intrauterine devices (IUDs). The findings revealed that women with two children were more likely to discontinue IUD use by 20.2%, whereas those with four children had a discontinuation rate of 16.5%. The utilization of IUDs is also influenced by the gender of children. The data indicate that 10.5% of women initiated IUD usage after having a male child, whereas only 5.8% of women did so after having a female child [16].

Vague Religious Beliefs and Justifications

Some individuals provide vague religious justifications for their refusal to utilize intrauterine devices (IUDs), citing religious prohibition as a basis. Such arguments posit that children are considered sacred blessings from a divine source, and therefore, denying them is deemed unacceptable [15]. Individuals often amalgamate their Islamic beliefs with their stance on the utilization of such contraceptive methods, perceiving it as a violation of their religious principles and considering it to be sinful [46]. Certain side effects, such as irregular menstruation, were also found to be the reason to quit IUDs because these were considered a hindrance in the way of completing ablutions and praying. In addition to the perceived somatic adverse effects associated with IUDs, there are also reported spiritual adverse effects such as spousal job loss, unproductive offspring, birth abnormalities in children, and even child mortality. These issues persist in the public domain and have not been adequately addressed [47].

Fear and Misconceptions Among Women Regarding IUDs

One of the primary obstacles to the uptake of intrauterine devices (IUDs) in young women is fear, which can manifest in various forms such as fear of pain during insertion, fear of device expulsion, and fear of potential physical damage caused by the device. This fear factor poses significant challenges to IUD placement among young women, potentially impacting their decision to use this contraceptive method [48]. Medical practitioners express concerns about patient safety, potential adverse effects, and overall patient satisfaction when it comes to IUDs. These considerations are significant factors that physicians take into account when recommending or prescribing IUDs as a contraceptive option [43]. The fear of experiencing dysmenorrhea, or menstrual pain, is recognized as a significant factor that deters women from either re-adopting or even considering the initiation of intrauterine devices (IUDs) as a contraceptive option [48]. Besides these, there are common misconceptions among individuals regarding the use of IUDs, with concerns including the potential for abortion, ectopic pregnancy, and discomfort. Additionally, there are fears that the IUD may rust inside the body and become displaced during sexual intercourse, leading to sexual complications [4,29].

Inaccessibility of Clients to IUD Facilities and High Cost of Services

The limited accessibility of contraceptives constitutes a significant impediment to their utilization as a family planning method and IUDs are at the top of all the methods because of their complexity of administration [49]. Poor health infrastructure and the unavailability of IUDs at many primary healthcare centers are contributing factors to the inaccessibility of clients to these facilities [50,51]. Low income and remote facilities are further creating hindrances in the access of the clients to the required facilities as more than 20% of the total population of Pakistan is below the poverty line [20,52,53]. The provision of family planning services, including IUDs, can be facilitated through hospital deliveries, but unfortunately, less than half of the total deliveries are in a hospital. A study conducted in Jhung revealed that 41% of women in the community delivered their babies in hospitals, but only 11% of them were utilizing family planning services [54].

Conceptualization of results

The aforementioned results are hereby summarized and categorized as per their relevance for easy and better understanding (Table 1):

**Table 1:** Summarization and conceptualization of results IUD: intrauterine device; IUCD: intrauterine contraceptive device

Health System Barriers	Users’ Barriers	Social and Religious Barriers	
Inadequate health care worker training. Lack of providers’ confidence. Governmental disinterest. Incomplete information and poor counseling on IUCDs.	High transport fares. Low economic status. Fears and misconceptions regarding IUDs.	Restrictions (like less mobility, Purdah, etc.) imposed on women. Refusal to use IUDs with social and religious prohibition as the basis though with vague justification.	
	Poor education and employment opportunities for women. Limited male partner involvement in Family Planning Programs. Aspirations for larger families and preference for male offspring. Illiteracy hindering understanding of IUDs.		
Post-insertion health concerns including pain and bleeding. Limited availability and accessibility of IUCD services in rural areas. Lack of client trust in health care providers due to inadequate training. Shortage of female health care providers. High costs of services.			Patriarchy limits women's say in family planning decisions. Irregular/heavy bleeding affects religious practices. Beliefs of spiritual losses and negative consequences from IUCDs.

Discussion

Our review is one of the first studies to highlight the barriers to the utilization of IUDs in Pakistan on account of society, government, and the health care system. In Pakistan, the most important impediments to the exercising of IUDs include the husband’s disagreement, social and cultural unacceptability, and anxiety and fear related to exaggerated side effects of using IUDs [14,55]. Illiteracy, poverty, and poor socioeconomic status also contributed to the restricted use of IUDs [56]. Other important factors contributing to such a low modern contraceptive prevalence rate of 35.4% in Pakistan include the conservative society imposing restrictions on women’s self-determination and self-governance. Due to purdah issues, it’s barely possible for many women in rural areas to leave their houses to seek knowledge from healthcare centers regarding IUDs [40]. Moreover, the distrust of the client the health worker who himself is not confident about providing adequate knowledge about usage, side effects, and long-term benefits of IUDs, also plays a key role [37]. Amalgamating religious thoughts with the practice of IUDs also creates hindrances [15,46].

A study showed that in 2012 there were 2.2 million abortions in Pakistan, making a total annual abortion rate of 50 in 1000 women. This abortion ratio explains the prevalence of unintended pregnancies occurring in society. Out of these women undergoing abortions, 623,000 women underwent postabortion complications [57]. This critical situation makes it the need of the hour to take serious action in this regard. Although several methods including rhythm, withdrawal, condoms, and sterilization are in practice, IUDs should be preferred for many reasons including reversibility of fertile life, easy insertion, no user dependency, and hideability, and now there are several international donor FP2020 programs that aim to provide cheaper services including IUDs [5,15,58]. But unfortunately, there are several barriers that hinder the use of IUDs to its maximum. The resolution to perform a particular action becomes a reality only if the hindrances in its way are removed and individuals have appropriate training for it [59,60]. The same is the case for IUDs. Several types of studies have been conducted throughout the world with similar results. A study conducted in Ghana confirms that the low prevalence of IUD utilization in women of Ghana is attributed to factors including uneasiness caused by placing IUDs, improper knowledge, and poor counseling [61].

Research done in India, Nigeria, and several other settings shows a pronounced negative association between nations that nurture a conservative cultural and religious belief system and IUCD usage [12,62,63]. Due to male dominance and family pressure faced by Asian women, many women find it hard to go out and opt for a family planning method [15,16,42]. Other low and middle-income countries have also observed a comparable pattern where the inadequacy of the use of IUDs is significantly impacted by the position of women and their partner's willingness to adopt contraception [64]. Misconceptions about the side effects of IUDs are considered an ominous hindrance to the continuous use of IUDs as illustrated by several studies conducted across the world [65-67]. The side effects explained by the users include fear of losing fertility, infections of the reproductive tract, and irregular menstruation [68]. A study regarding IUD acceptability showed there was a universal fear among adolescents about IUD placement. These fears were of different kinds such as fear of experiencing pain during the process of device insertion, fear of device discharge, and damage caused to the body by an IUD [69]. Insufficient confidence and training of health care workers is one of the prevailing hindrances in the utilization of IUDs worldwide due to various factors like PPIUDs training are carried out on anatomical models that are most often bulky, very expensive, and too fragile to be reused time and again. This also has limited the training session to large groups [70]. Consequently, there has been a decline in the number of healthcare providers who have received adequate training in PPIUD insertion, and even among those who have received training, there may be challenges in maintaining their proficiency due to various factors [71].

Despite extensive efforts made during this study, similar to any other research endeavor, certain limitations should be acknowledged. Firstly, non-English research articles were excluded from our analysis, and those without accessible full-text versions were also omitted. The exclusion of these articles could potentially impact our ability to identify variations and disparities. Additionally, although we utilized all the available resources, financial and official constraints prevented us from conducting a comprehensive search of grey literature or engaging in hand-searching. Furthermore, the studies included in our review exhibited diverse research methodologies and study designs, contributing to heterogeneity within the data. It is important to note that barriers to IUD utilization may evolve, and as such, our analysis may not fully capture such evolving differences. 

Recommendations

Enhance the understanding of the advantages of family planning and utilization of IUDs through awareness-raising initiatives. These initiatives should not solely concentrate on women but also include males within households as the secondary target audience. It is critical to engage both genders in activities that bring about behavioral change to tackle negative attitudes and low motivation toward the use of IUDs. The involvement of husbands and mothers-in-law is crucial in behavioral change campaigns. Additionally, it is imperative to improve the accessibility of healthcare centers that offer IUD services, particularly in remote areas that lack such facilities. Religious and community stakeholders should be involved to dispel any religious and cultural myths surrounding the use of IUDs.

## Conclusions

In this review, we have identified the prevailing impediments to the use of IUDs in Pakistan. These hindrances include a dearth of knowledge, insufficient motivation, resistance from husbands and in-laws, religious and cultural beliefs, restricted access, and communication barriers. It is imperative to increase awareness about IUDs as a modern contraceptive method amongst both men and women to enhance their comprehension and utilization of this method. Religious leaders and community stakeholders should take an active role in addressing the social factors that impact the use of IUDs.
